# One- and Two-Sample Predictions Based on Progressively Type-II Censored Carbon Fibres Data Utilizing a Probability Model

**DOI:** 10.1155/2022/6416806

**Published:** 2022-05-12

**Authors:** Mahmoud El-Morshedy, Rashad M. El-Sagheer, Samah H. El-Essawy, Khaled M. Alqahtani, Mohamed El-Dawoody, Mohamed S. Eliwa

**Affiliations:** ^1^Department of Mathematics, College of Science and Humanities in Al-Kharj, Prince Sattam Bin Abdulaziz University, Al-Kharj 11942, Saudi Arabia; ^2^Department of Mathematics, Faculty of Science, Mansoura University, Mansoura 35516, Egypt; ^3^Mathematics Department, Faculty of Science, Al-Azhar University, Naser 11884, Cairo, Egypt; ^4^Astronomy Department, National Research Institute of Astronomy and Geophysics, Naser 11884, Cairo, Egypt; ^5^Department of Statistics and Operation Research, College of Science, Qassim University, P.O. Box 6644, Buraydah 51482, Saudi Arabia; ^6^Department of Mathematics and Statistics, Faculty of Science, Mansoura University, Mansoura 35516, Egypt

## Abstract

New Weibull-Pareto distribution is a significant and practical continuous lifetime distribution, which plays an important role in reliability engineering and analysis of some physical properties of chemical compounds such as polymers and carbon fibres. In this paper, we construct the predictive interval of unobserved units in the same sample (one sample prediction) and the future sample based on the current sample (two-sample prediction). The used samples are generated from new Weibull-Pareto distribution due to a progressive type-II censoring scheme. Bayesian and maximum likelihood approaches are implemented to the prediction problems. In the Bayesian approach, it is not easy to simplify the predictive posterior density function in a closed form, so we use the generated Markov chain Monte Carlo samples from the Metropolis-Hastings technique with Gibbs sampling. Moreover, the predictive interval of future upper-order statistics is reported. Finally, to demonstrate the proposed methodology, both simulated data and real-life data of carbon fibres examples are considered to show the applicabilities of the proposed methods.

## 1. Introduction

Predictive analytics is used to reduce time, effort, and costs in forecasting business outcomes. A better decision will be supported when more data have been available. Moreover, organizations can solve their own problems and identify opportunities, by giving accurate and reliable insights. Using predictive analytics, we can analyse collective data to get new opportunities for customer attraction.

In the last few years, there has been growing interest in prediction which plays a vital role in many fields. For example, in industry, the experimenter wants to predict the lifetime of a future unobserved unit that relies on the information available from the current sample. So, the experimenter or the manufacturer introduces its products in the market and wants to make it on the place of desire and the focus of consumers by making their warranty limits more acceptable to them. For more information about applications of prediction, the reader can see the following researches: Ghafouri et al. [1], Pushpalatha et al. [2], Lee et al. [3], Burnaev [4], Sharma and Vijayakumar [5], and Asher et al. [6].

**Figure 1:**
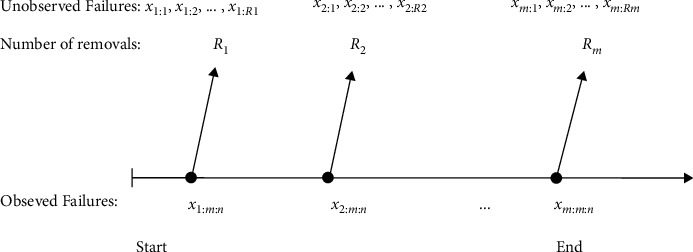
Diagram of OSP under progT-II CS.

The future prediction problem can be separated into two types as follows: the first type is known as an OSP problem, and the other one is a TSP problem. In the OSP problem, the variable to be predicted comes from the same sequence of variables observed and is dependent on the current sample (see Figure 1). In the second type, the variable to be predicted comes from another independent future sample.

Suleman and Albert [7] suggested a new generalization form of Weibull-Pareto distribution denoted by NWPD, which is useful in modeling real-life situations and different scientific disciplines fields such as biological and marketing science in addition to reliability analysis and life testing. The probability density function (pdf) and cumulative distribution function (cdf) of a random variable *X* having an NWPD which is denoted by NWPD (*δ*, *β*, *θ*) are given, respectively, by

(1) $$\begin{array}{c} f\left(x;\delta ,\beta ,\theta \right)=\frac{\beta \delta }{\theta }{\left(\frac{x}{\theta }\right)}^{\beta -1}e^{-\delta {\left(x/\theta \right)}^{\beta }},\;x>\text{0};\;\delta ,\beta ,\theta >\text{0,} \end{array}$$

(2) $$\begin{array}{c} F\left(x;\delta ,\beta ,\theta \right)=1-e^{-\delta {\left(x/\theta \right)}^{\beta }},\;x>\text{0};\;\delta ,\beta ,\theta >\text{0}, \end{array}$$

where *δ* and *θ* are the scale parameters, and *β* is the shape parameter. The reliability function *S*(*t*) and hazard rate function *h*(*t*) of the NWPD take the forms, respectively, as

(3) $$\begin{array}{c} S\left(t\right)=e^{-\delta {\left(t/\theta \right)}^{\beta }},\;t>\text{0,} \end{array}$$

(4) $$\begin{array}{c} h\left(t\right)=\frac{\beta \delta }{\theta }{\left(\frac{t}{\theta }\right)}^{\beta -1},\;t>\text{0}. \end{array}$$

It should be noted that the NWPD reduces to well-known distributions such as Weibull, Rayleigh, and exponential distributions as follows:

- If *δ*=*θ*=1, then NWPD reduces to Weibull (*β*, 1).
- If *δ*=1, then NWPD reduces to Weibull (*β*, *θ*).
- If *δ*=1/2 and *β*=2, then NWPD reduces to Rayleigh (*θ*).
- If *β*=1, then NWPD reduces to an exponential distribution with a mean equal *θ*/*δ*.

It is clear that the shape of *h*(*t*) depends on the parameter *β*, and the following can be observed.

**Figure 2:**
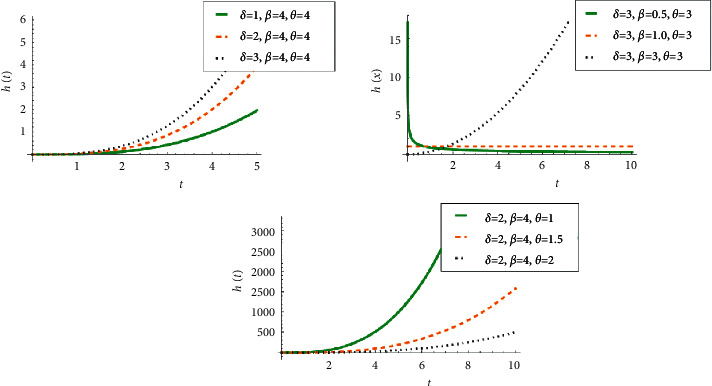
The hazard rate function of NWPD for different values of *δ*, *β*, and *θ*.

- If *β*=1, the failure rate is constant and given by *h*(*t*)=*δ*/*θ*. This makes the NWPD suitable for modeling systems or components with a constant failure rate.
- If *β* > 1, the hazard is an increasing function of *t*, which makes the NWPD suitable for modeling components that wear faster with time.
- If *β* < 1, the hazard is a decreasing function of *t*, which makes the NWPD suitable for modeling components that wear slower with time. For a quick illustration, see Figure 2.

The designed body of the paper is built to obtain the Bayesian and frequentist prediction under a ProgT-II C sample whose lifetime failures have NWPD. We study two popular techniques of the prediction problems known as OSP and TSP. As a vivid example of the applicability of the methodology used in our paper, the new Weibull-Pareto distribution was applied to model the exceedances of flood peaks (in m^3^/s) of the Wheaton River near Carcross in Yukon Territory, Canada. In our paper, in the case of a one-sample prediction, it is possible to predict the values of the exceedances of the flood peaks that were not recorded for any reason while, in the case two-sample prediction, it is possible to predict the excesses of future flood peaks based on the available data. Accordingly, the necessary precautions can be taken to limit the destruction that may be caused by the flood. There are several kinds of literature discussing the prediction problem under the ProgT-II CS for different distributions, for instance, Ghafouri et al. [8], Abdel-Hamid [9], AL-Hussaini et al. [10], Raqab et al. [11], Golparvar and Parsian [12] and Soliman et al. [13].

Also, many authors have focused on the problem of predicting either TSP or OSP and TSP together based on various types of censored data from different lifetime models, see, for example, Mahmoud et al. [14], EL-Sagheer [15], Ahmed [16], and Abushal and Al-Zaydi [17, 18].

The remainder of the paper is organized as follows: the ML and Bayesian point estimates of the unknown parameters are discussed in Section 2. In Section 3, the MLPI and BPI are explained in the case of OSP. The MLPI and BPI of the FOS sample are outlined in Section 4. In the same section, the MLPI and BPI for the FURS sample are also obtained.  Section 5 is devoted to analyse two real-life examples. Conclusion remarks and the results of this work are reported in Section 6.

## 2. Maximum Likelihood and Bayesian Approaches

Suppose that *X*_1:*m*:*n*_^(*R*_1_,…,*R*_*m*_)^, *X*_2:*m*:*n*_^(*R*_1_,…,*R*_*m*_)^,…, *X*_*m*:*m*:*n*_^(*R*_1_,…,*R*_*m*_)^ be a ProgT-II C sample from the NWPD with a progressive censored scheme *R*=(*R*_1_, *R*_2_,…, *R*_*m*_). According to Balakrishnan and Aggarwala [19], the joint probability density function is given by

(5) $$\begin{array}{c} f_{1,2,\ldots ,m}\left(x_{1:m:n}^{\left(R_{1},\ldots ,R_{m}\right)},x_{2:m:n}^{\left(R_{1},\ldots ,R_{m}\right)},\ldots ,x_{m:m:n}^{\left(R_{1},\ldots ,R_{m}\right)}\right)\propto \prod_{i=1}^{m}f\left(x_{i:m:n}^{\left(R_{1},\ldots ,R_{m}\right)}\right){\left[1-F\left(x_{i:m:n}^{\left(R_{1},\ldots ,R_{m}\right)}\right)\right]}^{R_{i}}. \end{array}$$

Inserting (1) and (2) into (5), then the likelihood function can be written as

(6) $$\begin{array}{c} L\left(\underline{x};\delta ,\beta ,\theta \right)\propto \beta^{m}\delta^{m}\theta^{\left(-m\right)}\left[\prod_{i=1}^{m}{\left(\frac{x_{i}}{\theta }\right)}^{\beta -1}\right]e^{\left\{-\delta \sum_{i=1}^{m}\left(R_{i}+1\right){\left(x_{i}/\theta \right)}^{\beta }\right\}}. \end{array}$$

Therefore, the log-likelihood function $\ell \left(\underline{x};\delta ,\beta ,\theta \right)$ can be expressed as

(7) $$\begin{array}{c} \ell \left(\underline{x};\delta ,\beta ,\theta \right)=m\;\log\left[\beta \right]+m\;\log\left[\delta \right]-m\;\log\left[\theta \right]+\left(\beta -1\right)\sum_{i=1}^{m}\log\left[\frac{x_{i}}{\theta }\right]-\delta \sum_{i=1}^{m}\left(R_{i}+1\right){\left(\frac{x_{i}}{\theta }\right)}^{\beta }. \end{array}$$

Upon differentiating (7) with respect to *δ*, *β*, and *θ*, respectively, and equating each result to zero, we obtain

(8) $$\begin{array}{c} \frac{m}{\delta }-\sum_{i=1}^{m}\left(R_{i}+1\right){\left(\frac{x_{i}}{\theta }\right)}^{\beta }=0, \end{array}$$

(9) $$\begin{array}{c} \frac{m}{\beta }+\sum_{i=1}^{m}\log\left[\frac{x_{i}}{\theta }\right]-\delta \sum_{i=1}^{m}\left(R_{i}+1\right){\left(\frac{x_{i}}{\theta }\right)}^{\beta }\log\left[\frac{x_{i}}{\theta }\right]=0, \end{array}$$

(10) $$\begin{array}{c} \frac{m\beta }{\theta }-\frac{\beta \delta }{\theta }\sum_{i=1}^{m}\left(R_{i}+1\right){\left(\frac{x_{i}}{\theta }\right)}^{\beta }=0. \end{array}$$

From (8), we get MLE of *δ* as

(11) $$\begin{array}{c} \hat{\delta }=m{\left[\sum_{i=1}^{m}\left(R_{i}+1\right){\left(\frac{x_{i}}{\hat{\theta }}\right)}^{\hat{\beta }}\right]}^{-1}. \end{array}$$

Since (9) and (10) do not have closed-form solutions, the Newton-Raphson iteration method can be used to get the MLEs of *β* and *θ*. The reader can see the detailed steps of the Newton-Raphson algorithm in EL-Sagheer [20]. Now, we discuss how to obtain the Bayesian estimates for *δ*, *β*, and *θ*. Let the parameters *δ*, *β*, and *θ* be independent and follow the gamma prior distributions as

(12) $$\begin{array}{c} \left.\begin{array}{c} \pi_{1}\left(\delta \right)=\frac{\eta_{1}^{\gamma_{1}}}{\Gamma \left(\gamma_{1}\right)}\delta^{\gamma_{1}-1}e^{-\eta_{1}\delta },\delta >\text{0},\gamma_{1}>\text{0},\eta_{1}>\text{0},\\ \pi_{2}\left(\beta \right)=\frac{\eta_{2}^{\gamma_{2}}}{\Gamma \left(\gamma_{2}\right)}\beta^{\gamma_{2}-1}e^{-\eta_{2}\beta },\;\beta >\text{0},\gamma_{2}>\text{0},\eta_{2}>\text{0},\\ \pi_{3}\left(\theta \right)=\frac{\eta_{3}^{\gamma_{3}}}{\Gamma \left(\gamma_{3}\right)}\theta^{\gamma_{3}-1}e^{-\eta_{3}\theta },\theta >\text{0},\gamma_{3}>\text{0},\eta_{3}>\text{0}. \end{array}\right\}, \end{array}$$

where the hyperparameters *γ*_*i*_ and *η*_*i*_ (where *i*=1,2,3) are reflected prior knowledge about *δ*, *β*, and *θ*. Note if *γ*_*i*_=*η*_*i*_=0, then the noninformative priors of *δ*, *β*, and *θ* are obtained.

Hence, the joint prior function of the parameters *δ*, *β*, and *θ* is defined by

(13) $$\begin{array}{c} \pi \left(\delta ,\beta ,\theta \right)=\frac{\eta_{1}^{\gamma_{1}}\eta_{2}^{\gamma_{2}}\eta_{3}^{\gamma_{3}}}{\Gamma \left(\gamma_{1}\right)\Gamma \left(\gamma_{2}\right)\Gamma \left(\gamma_{3}\right)}\delta^{\gamma_{1}-1}\beta^{\gamma_{2}-1}\theta^{\gamma_{3}-1}e^{-\eta_{1}\delta -\eta_{2}\beta -\eta_{3}\theta }. \end{array}$$

From (6) and (13), the joint posterior density function can be given as follows:

(14) $$\begin{array}{c} \pi^{*}\left(\delta ,\beta ,\theta |\underline{x}\right)=\frac{L\left(\underline{x};\delta ,\beta ,\theta \right)\times \pi \left(\delta ,\beta ,\theta \right)}{\int_{0}^{\infty }\int_{0}^{\infty }\int_{0}^{\infty }L\left(\underline{x};\delta ,\beta ,\theta \right)\times \pi \left(\delta ,\beta ,\theta \right)\text{d}\delta \text{d}\beta \text{d}\theta }\\ \propto \beta^{m+\gamma_{2}-1}\delta^{m+\gamma_{1}-1}\theta^{\left(-m+\gamma_{3}-1\right)}\left[\prod_{i=1}^{m}{\left(\frac{x_{i}}{\theta }\right)}^{\beta -1}\right]\\ \times e^{\left\{-\eta_{2}\beta -\eta_{3}\theta -\delta \left[\eta_{1}+\sum_{i=1}^{m}R_{i}+1{\left(x_{i}/\theta \right)}^{\beta }\right]\right\}}. \end{array}$$

It is clear that (14) cannot be obtained in a closed form. So, we apply the M-H technique with Gibbs sampling to generate MCMC samples and obtain the Bayes estimates of *δ*, *β*, and *θ*. The reader can see the detailed steps of the M-H technique with Gibbs sampling in the study of Mahmoud et al. [21].

## 3. One-Sample Prediction

OSP is a useful method to predict the failure lifetimes of the unobserved units (the removed surviving units) in the same sample generated by the ProgT-II C sample *X*_1:*m*:*n*_^(*R*_1_,…,*R*_*m*_)^, *X*_2:*m*:*n*_^(*R*_1_,…,*R*_*m*_)^ ,…, *X*_*m*:*m*:*n*_^(*R*_1_,…,*R*_*m*_)^ with a progressive censoring scheme (*R*_1_, *R*_2_,…, *R*_*m*_). Suppose that *X*_*i*:*R*_*l*__, *i*=1,2,…, *R*_*l*_ and *l*=1,2,…, *m* denote failure lifetimes of *i*^th^ unobserved units, then the conditional pdf of *X*_*i*:*R*_*l*__ ≡ *x*_*i*:*R*_*l*__ for a given value of *δ*, *β*, and *θ* defined as

(15) $$\begin{array}{c} g_{1}\left(x_{i:R_{l}}|\delta ,\beta ,\theta ,\underline{x}\right)=i\left(\begin{array}{c} R_{l}\\ i \end{array}\right){\left[F\left(x_{i:R_{l}};\delta ,\beta ,\theta \right)-F\left(x;\delta ,\beta ,\theta \right)\right]}^{i-1}{\left[1-F\left(x_{i:R_{l}};\delta ,\beta ,\theta \right)\right]}^{R_{l}-i}\\ \times f\left(x_{i:R_{l}};\delta ,\beta ,\theta \right){\left[1-F\left(x;\delta ,\beta ,\theta \right)\right]}^{-R_{l}},\;x_{i:R_{l}}>x. \end{array}$$

Inserting (1) and (2) in (13), we get

(16) $$\begin{array}{c} g_{1}\left(x_{i:R_{l}}|\delta ,\beta ,\theta ,\underline{x}\right)=i\left(\begin{array}{c} R_{l}\\ i \end{array}\right)\frac{\beta \delta }{\theta }{\left(\frac{x_{i:R_{l}}}{\theta }\right)}^{\beta -1}e^{R_{l}\delta {\left(x/\theta \right)}^{\beta }}e^{-\left(R_{l}-i+1\right)\delta {\left(x_{i:R_{l}}/\theta \right)}^{\beta }}{\left[e^{-\delta {\left(x/\theta \right)}^{\beta }}-e^{-\delta {\left(x_{i:R_{l}}/\theta \right)}^{\beta }}\right]}^{i-1}\\ =i\left(\begin{array}{c} R_{l}\\ i \end{array}\right)\frac{\beta \delta }{\theta }{\left(\frac{x_{i:R_{l}}}{\theta }\right)}^{\beta -1}\sum_{k=0}^{i-1}\left(\begin{array}{c} i-1\\ k \end{array}\right){\left(-1\right)}^{k}e^{-\left(R_{l}-i+k+1\right)\delta {\left(x_{i:R_{l}}/\theta \right)}^{\beta }}\\ \times e^{-\left(-R_{l}+i-k-1\right)\delta {\left(x/\theta \right)}^{\beta }}. \end{array}$$

The distribution function of *x*_*i*:*R*_*l*__ can be defined by

(17) $$\begin{array}{c} G_{1}\left(x_{i:R_{l}}|\delta ,\beta ,\theta ,\underline{x}\right)=\int_{x}^{x_{i:R_{l}}}g_{1}\left(x_{i:R_{l}}|\delta ,\beta ,\theta ,\underline{x}\right)\text{d}x_{i:R_{l}}\\ =i\left(\begin{array}{c} R_{l}\\ i \end{array}\right)\sum_{k=0}^{i-1}\left(\begin{array}{c} i-1\\ k \end{array}\right){\left(-1\right)}^{k}e^{-\left(-R_{l}+i-k-1\right)\delta {\left(x/\theta \right)}^{\beta }}\\ \times \left(\frac{e^{-\left(R_{l}-i+k+1\right)\delta {\left(x/\theta \right)}^{\beta }}-e^{-\left(R_{l}-i+k+1\right)\delta {\left(x_{i:R_{l}}/\theta \right)}^{\beta }}}{\left(R_{l}-i+k+1\right)}\right). \end{array}$$

### 3.1. Maximum Likelihood Prediction

Due to ML prediction, the (1 − *γ*)100% MLPI (LB_1_,UB_1_) of *x*_*i*:*R*_*l*__ can be written in the form

(18) $$\begin{array}{c} \text{Pr}\left[x_{i:R_{l}}>{\text{LB}}_{1}|\underline{x}\right]=1-\frac{\gamma }{2}=1-{\hat{G}}_{1}\left({\text{LB}}_{1}|\underline{x}\right)\Rightarrow {\hat{G}}_{1}\left({\text{LB}}_{1}|\underline{x}\right)=\frac{\gamma }{2}, \end{array}$$

(19) $$\begin{array}{c} \text{Pr}\left[x_{i:R_{l}}>{\text{UB}}_{1}|\underline{x}\right]=\frac{\gamma }{2}=1-{\hat{G}}_{1}\left({\text{UB}}_{1}|\underline{x}\right)\Rightarrow {\hat{G}}_{1}\left({\text{UB}}_{1}|\underline{x}\right)=1-\frac{\gamma }{2}, \end{array}$$

where ${\hat{G}}_{1}\left(x_{i:R_{l}}|\underline{x}\right)$ can be obtained after replacing the values of *δ*, *β*, and *θ* by their point estimates $\hat{\delta },\hat{\beta }$, and $\hat{\theta }$ as in (17). Newton-Raphson iteration method is employed to get the approximated solutions of (18) and (19).

### 3.2. Bayesian Prediction

Using (14) and (16), the predictive posterior density function of *x*_*i*:*R*_*l*__ be given in the following form:

(20) $$\begin{array}{c} g_{1}^{*}\left(x_{i:R_{l}}|\underline{x}\right)=\int_{0}^{\infty }\int_{0}^{\infty }\int_{0}^{\infty }g_{1}\left(x_{i:R_{l}}|\delta ,\beta ,\theta ,\underline{x}\right)\pi^{*}\left(\delta ,\beta ,\theta |\underline{x}\right)\text{d}\delta \text{d}\beta \text{d}\theta . \end{array}$$

It is so hard to simplify (20) in a closed formula. So, MCMC samples generated by applying the M-H technique within Gibbs sampling can be used to approximate the $g_{1}^{*}\left(x_{i:R_{l}}|\underline{x}\right)$ as

(21) $$\begin{array}{c} {\hat{g}}_{1}^{*}\left(x_{i:R_{l}}|\underline{x}\right)=\frac{1}{N-M}\sum_{j=M+1}^{N}g_{1}\left(x_{i:R_{l}}|\delta_{j},\beta_{j},\theta_{j},\underline{x}\right). \end{array}$$

As in (17), we can approximate the distribution function of *x*_*i*:*R*_*l*__ based on the generated MCMC samples as follows:

(22) $$\begin{array}{c} {\hat{G}}_{1}^{*}\left(x_{i:R_{l}}|\underline{x}\right)=\frac{1}{N-M}\sum_{j=M+1}^{N}G_{1}\left(x_{i:R_{l}}|\delta_{j},\beta_{j},\theta_{j},\underline{x}\right). \end{array}$$

Then, the (1 − *γ*)100% BPI (LB_1_,UB_1_) of *x*_*i*:*R*_*l*__ takes the form as

(23) $$\begin{array}{c} \text{Pr}\left[x_{i:R_{l}}>{\text{LB}}_{1}|\underline{x}\right]=1-\frac{\gamma }{2}=1-{\hat{G}}_{1}^{*}\left({\text{LB}}_{1}|\underline{x}\right)\Rightarrow {\hat{G}}_{1}^{*}\left({\text{LB}}_{1}|\underline{x}\right)=\frac{\gamma }{2}, \end{array}$$

(24) $$\begin{array}{c} \text{Pr}\left[x_{i:R_{l}}>{\text{UB}}_{1}|\underline{x}\right]=\frac{\gamma }{2}=1-{\hat{G}}_{1}^{*}\left({\text{UB}}_{1}|\underline{x}\right)\Rightarrow {\hat{G}}_{1}^{*}\left({\text{UB}}_{1}|\underline{x}\right)=1-\frac{\gamma }{2}. \end{array}$$

To solve (23) and (24), we employ the Newton-Raphson iteration method.

## 4. Two-Sample Prediction

TSP is a useful method to predict the failure lifetimes in the future sample based on the available current sample which was drawn from the same population. In this section, we discuss two cases of TSP. The first one is the TSP for FOS, and the other is the TSP for FURS. Also, the construction of PI based on ML and Bayesian predictions in the two cases of TSP is discussed.

### 4.1. Prediction of Future-Order Statistics

Suppose that the available current sample *X*_1:*m*:*n*_^(*R*_1_,…,*R*_*m*_)^, *X*_2:*m*:*n*_^(*R*_1_,…,*R*_*m*_)^,…, *X*_*m*:*m*:*n*_^(*R*_1_,…,*R*_*m*_)^ be a ProgT-II C sample and let *Y*_1_, *Y*_2_,…, *Y*_*n*_1__ be the FOS sample drawn from the same NWPD (*δ*, *β*, *θ*). Our concern is to make predictions about the *s*^th^, 1 ≤ *s* ≤ *n*_1_ FOS values. The conditional pdf of FOS *Y*_*s*_ for a given values of *δ*, *β*, and *θ* is expressed in the formula, see David and Nagaraja [22].

(25) $$\begin{array}{c} g_{2}\left(y_{s}|\delta ,\beta ,\theta ,\underline{x}\right)=s\left(\begin{array}{c} n_{1}\\ s \end{array}\right){\left[1-F\left(y_{s};\delta ,\beta ,\theta \right)\right]}^{n_{1}-s}{\left[F\left(y_{s};\delta ,\beta ,\theta \right)\right]}^{s-1}f\left(y_{s};\delta ,\beta ,\theta \right). \end{array}$$

Inserting (1) and (2) in (23), we get

(26) $$\begin{array}{c} g_{2}\left(y_{s};\delta ,\beta ,\theta \right)=s\left(\begin{array}{c} n_{1}\\ s \end{array}\right)\frac{\beta \delta }{\theta }{\left(\frac{y_{s}}{\theta }\right)}^{\beta -1}e^{-\left(n_{1}-s+1\right)\delta {\left(y_{s}/\theta \right)}^{\beta }}{\left[1-e^{-\delta {\left(y_{s}/\theta \right)}^{\beta }}\right]}^{s-1}\\ =s\left(\begin{array}{c} n_{1}\\ s \end{array}\right)\frac{\beta \delta }{\theta }{\left(\frac{y_{s}}{\theta }\right)}^{\beta -1}\sum_{k=0}^{s-1}\left(\begin{array}{c} s-1\\ k \end{array}\right){\left(-1\right)}^{k}e^{-\left(n_{1}-s+k+1\right)\delta {\left(y_{s}/\theta \right)}^{\beta }}. \end{array}$$

The distribution function of *Y*_*s*_ takes the form

(27) $$\begin{array}{c} G_{2}\left(y_{s}|\delta ,\beta ,\theta ,\underline{x}\right)=\int_{0}^{y_{s}}g_{2}\left(y_{s}|\delta ,\beta ,\theta ,\underline{x}\right){\text{dy}}_{s}\\ =s\left(\begin{array}{c} n_{1}\\ s \end{array}\right)\sum_{k=0}^{s-1}\left(\begin{array}{c} s-1\\ k \end{array}\right){\left(-1\right)}^{k}\left(\frac{1-e^{-\left(n_{1}-s+k+1\right)\delta {\left(y_{s}/\theta \right)}^{\beta }}}{\left(n_{1}-s+k+1\right)}\right). \end{array}$$

#### 4.1.1. Maximum Likelihood Prediction

Due to ML prediction, PI of FOS *y*_*s*_ can be computed by replacing the values of *δ*, *β*, and *θ* by their point estimates $\hat{\delta },\hat{\beta }$, and $\hat{\theta }$. The (1 − *γ*)100% MLPI (LB_2_,UB_2_) of FOS *y*_*s*_ takes the form as

(28) $$\begin{array}{c} \text{Pr}\left[y_{s}>{\text{LB}}_{2}|\underline{x}\right]=1-\frac{\gamma }{2}=1-{\hat{G}}_{2}\left({\text{LB}}_{2}|\underline{x}\right)\Rightarrow {\hat{G}}_{2}\left({\text{LB}}_{2}|\underline{x}\right)=\frac{\gamma }{2}, \end{array}$$

(29) $$\begin{array}{c} \text{Pr}\left[y_{s}>{\text{UB}}_{2}|\underline{x}\right]=\frac{\gamma }{2}=1-{\hat{G}}_{2}\left({\text{UB}}_{2}|\underline{x}\right)\Rightarrow {\hat{G}}_{2}\left({\text{UB}}_{2}|\underline{x}\right)=1-\frac{\gamma }{2}. \end{array}$$

It is evident that (28) and (29) do not have an analytical solution; therefore, the Newton-Raphson iteration method is applied to get the approximated solutions.

#### 4.1.2. Bayesian Prediction

The predictive posterior density function of FOS *y*_*s*_ can be written using (14) and (29) as follows:

(30) $$\begin{array}{c} g_{2}^{*}\left(y_{s}|\underline{x}\right)=\int_{0}^{\infty }\int_{0}^{\infty }\int_{0}^{\infty }g_{2}\left(y_{s}|\delta ,\beta ,\theta ,\underline{x}\right)\pi^{*}\left(\delta ,\beta ,\theta |\underline{x}\right)\text{d}\delta \text{d}\beta \text{d}\theta . \end{array}$$

The approximated solution of $g_{2}^{*}\left(y_{s}|\underline{x}\right)$ and its distribution function can be obtained by applying the generated MCMC samples as follows:

(31) $$\begin{array}{c} {\hat{g}}_{2}^{*}\left(y_{s}|\underline{x}\right)=\frac{1}{N-M}\sum_{j=M+1}^{N}g_{1}\left(y_{s}|\delta_{j},\beta_{j},\theta_{j},\underline{x}\right), \end{array}$$

(32) $$\begin{array}{c} {\hat{G}}_{2}^{*}\left(y_{s}|\underline{x}\right)=\frac{1}{N-M}\sum_{j=M+1}^{N}G_{2}\left(y_{s}|\delta_{j},\beta_{j},\theta_{j},\underline{x}\right). \end{array}$$

Therefore, the (1 − *γ*)100% BPI (LB_2_,UB_2_) of FOS *y*_*s*_ is constructed.

(33) $$\begin{array}{c} \text{Pr}\left[y_{s}>{\text{LB}}_{2}|\underline{x}\right]=1-\frac{\gamma }{2}=1-{\hat{G}}_{2}^{*}\left({\text{LB}}_{2}|\underline{x}\right)\Rightarrow {\hat{G}}_{2}^{*}\left({\text{LB}}_{2}|\underline{x}\right)=\frac{\gamma }{2}, \end{array}$$

(34) $$\begin{array}{c} \text{Pr}\left[y_{s}>{\text{UB}}_{2}|\underline{x}\right]=\frac{\gamma }{2}=1-{\hat{G}}_{2}^{*}\left({\text{UB}}_{2}|\underline{x}\right)\Rightarrow {\hat{G}}_{2}^{*}\left({\text{UB}}_{2}|\underline{x}\right)=1-\frac{\gamma }{2}. \end{array}$$

We need to apply some suitable numerical techniques such Newton-Raphson iteration method for solving (33) and (34).

### 4.2. Prediction of Future Upper Record Statistics

Suppose that the available current sample *X*_1:*m*:*n*_^(*R*_1_,…,*R*_*m*_)^, *X*_2:*m*:*n*_^(*R*_1_,…,*R*_*m*_)^,…, *X*_*m*:*m*:*n*_^(*R*_1_,…,*R*_*m*_)^ be ProgT-II C sample and let *Z*_*U*(1)_, *Z*_*U*(2)_,…, *Z*_*U*(*n*_2_)_ be the FURS sample drawn from the same NWPD (*δ*, *β*, *θ*). We want to make predictions about the *s*^th^, 1 ≤ *s* ≤ *n*_2_ FURS values. The conditional pdf of FURS *Z*_*s*_ for a given value of *δ*, *β*, and *θ* is given in the form; see Chandler [23].

(35) $$\begin{array}{c} g_{3}\left(z_{s}|\delta ,\beta ,\theta ,\underline{x}\right)=\frac{1}{\left(s-1\right)!}{\left\{-\log\left[1-F\left(z_{s};\delta ,\beta ,\theta \right)\right]\right\}}^{s-1}f\left(z_{s};\delta ,\beta ,\theta \right). \end{array}$$

Inserting (1) and (2) in (33), we get

(36) $$\begin{array}{c} g_{3}\left(z_{s}|\delta ,\beta ,\theta ,\underline{x}\right)=\frac{1}{\left(s-1\right)!}\frac{\beta \delta }{\theta }{\left(\frac{z_{s}}{\theta }\right)}^{\beta -1}{\left\{-\log\left[1-\left(1-e^{-\delta {\left(z_{s}/\theta \right)}^{\beta }}\right)\right]\right\}}^{s-1}e^{-\delta {\left(z_{s}/\theta \right)}^{\beta }}. \end{array}$$

The distribution function of *Z*_*s*_ defined as follows:

(37) $$\begin{array}{c} G_{3}\left(z_{s}|\delta ,\beta ,\theta ,\underline{x}\right)=\int_{0}^{z_{s}}g_{3}\left(z_{s}|\delta ,\beta ,\theta ,\underline{x}\right)\text{d}z_{s}\\ =\frac{1}{\left(s-1\right)!}\frac{\beta \delta }{\theta }\int_{0}^{z_{s}}{\left(\frac{z_{s}}{\theta }\right)}^{\beta -1}{\left\{-\log\left[1-\left(1-e^{-\delta {\left(z_{s}/\theta \right)}^{\beta }}\right)\right]\right\}}^{s-1}e^{-\delta {\left(z_{s}/\theta \right)}^{\beta }}\text{d}z_{s}\\ =\frac{1}{\left(s-1\right)!}\int_{0}^{1-\left(1-e^{-\delta {\left(z_{s}/\theta \right)}^{\beta }}\right)}{\left\{-\log\left[\varphi \right]\right\}}^{s-1}\text{d}\varphi \\ =\frac{1}{\left(s-1\right)!}\left(\Gamma \left[s\right]-\Gamma \left[s,-\log\left[1-\left(1-e^{-\delta {\left(z_{s}/\theta \right)}^{\beta }}\right)\right]\right]\right). \end{array}$$

#### 4.2.1. Maximum Likelihood Prediction

Due to ML prediction, PI of *z*_*s*_ can be computed by replacing the values of *δ*, *β*, and *θ* by their point estimates $\hat{\delta },\hat{\beta }$, and $\hat{\theta }$. The (1 − *γ*)100% MLPI (LB_3_,UB_3_) of FURS *z*_*s*_ takes the form as

(38) $$\begin{array}{c} \text{Pr}\left[z_{s}>{\text{LB}}_{3}|\underline{x}\right]=1-\frac{\gamma }{2}=1-{\hat{G}}_{3}\left({\text{LB}}_{3}|\underline{x}\right)\Rightarrow {\hat{G}}_{3}\left({\text{LB}}_{3}|\underline{x}\right)=\frac{\gamma }{2}, \end{array}$$

(39) $$\begin{array}{c} \text{Pr}\left[z_{s}>{\text{UB}}_{3}|\underline{x}\right]=\frac{\gamma }{2}=1-{\hat{G}}_{3}\left({\text{UB}}_{3}|\underline{x}\right)\Rightarrow {\hat{G}}_{3}\left({\text{UB}}_{3}|\underline{x}\right)=1-\frac{\gamma }{2}. \end{array}$$

For solving (38) and (39), we use the Newton-Raphson iteration method.

#### 4.2.2. Bayesian Prediction

The predictive posterior density function of FURS *z*_*s*_ can be written using (14) and (36) as follows:

(40) $$\begin{array}{c} g_{3}^{*}\left(z_{s}|\underline{x}\right)=\int_{0}^{\infty }\int_{0}^{\infty }\int_{0}^{\infty }g_{3}\left(z_{s}|\delta ,\beta ,\theta ,\underline{x}\right)\pi^{*}\left(\delta ,\beta ,\theta |\underline{x}\right)\text{d}\delta \text{d}\beta \text{d}\theta . \end{array}$$

The approximated solution of $g_{3}^{*}\left(z_{s}|\underline{x}\right)$ and its distribution function can be obtained by applying the generated MCMC samples as follows:

(41) $$\begin{array}{c} {\hat{g}}_{3}^{*}\left(z_{s}|\underline{x}\right)=\frac{1}{N-M}\sum_{j=M+1}^{N}g_{3}\left(z_{s}|\delta_{j},\beta_{j},\theta_{j},\underline{x}\right), \end{array}$$

(42) $$\begin{array}{c} {\hat{G}}_{3}^{*}\left(z_{s}|\underline{x}\right)=\frac{1}{N-M}\sum_{j=M+1}^{N}G_{3}\left(z_{s}|\delta_{j},\beta_{j},\theta_{j},\underline{x}\right). \end{array}$$

Therefore, the (1 − *γ*)100% BPI (LB_3_,UB_3_) of FURS *z*_*s*_ can be obtained in the following form:

(43) $$\begin{array}{c} \text{Pr}\left[z_{s}>{\text{LB}}_{3}|\underline{x}\right]=1-\frac{\gamma }{2}=1-{\hat{G}}_{3}^{*}\left({\text{LB}}_{3}|\underline{x}\right)\Rightarrow {\hat{G}}_{3}^{*}\left({\text{LB}}_{3}|\underline{x}\right)=\frac{\gamma }{2}, \end{array}$$

(44) $$\begin{array}{c} \text{Pr}\left[z_{s}>{\text{UB}}_{3}|\underline{x}\right]=\frac{\gamma }{2}=1-{\hat{G}}_{3}^{*}\left({\text{UB}}_{3}|\underline{x}\right)\Rightarrow {\hat{G}}_{3}^{*}\left({\text{UB}}_{3}|\underline{x}\right)=1-\frac{\gamma }{2}. \end{array}$$

We need to apply some suitable numerical techniques such Newton-Raphson iteration method for solving (43) and (44).

## 5. Numerical Computations

To illustrate the proposed methods discussed in the previous sections, we consider two examples, the first one is a simulated data set, and the other is a real data set.

**Example 1 .**

(Simulated data). Based on the algorithm which is introduced by Balakrishnan and Sandhu [24], we generate a ProgT-II C sample from NWPD with parameters (*δ*, *β*, *θ*)=(2.4, 1.8, 2.9) of size *m*=30, which is generated randomly of sample size *n*=50 with censoring scheme *R* = (2, 0, 0, 1, 0, 0, 2, 0, 0, 2, 0, 1, 0, 0, 2, 0, 0, 0, 0, 2, 0, 0, 2, 0, 2, 0, 2, 0, 0. 2). The ProgT-II C sample is given *x* = (0.205494, 0.274422, 0.360082, 0.416501, 0.527163, 0.58346, 0.614485, 0.665395, 0.666271, 0.693925, 0.697056, 0.893878, 0.920077, 0.929093, 0.956805, 0.978055, 1.11192, 1.27356, 1.3368, 1.35507, 1.38305, 1.59598, 1.63893, 1.86817, 1.90648, 2.01795, 2.02848, 2.2878, 2.37404, 2.51562). Based on the M-H technique within Gibbs sampling, we generate 32000 MCMC samples {(*δ*_*j*_, *β*_*j*_, *θ*_*j*_), *j*=1,2,…, 32000} and discard the first 2000 values as “burn-in” periods under the consideration of the noninformative prior gamma functions of *δ*, *β*, and *θ* with hyperparameters *γ*_*i*_ and *η*_*i*_=0, where *i*=1,2,3. The mean values of *δ*, *β*, and *θ* are given in Table 1. The results of 90% MLPI and BPI of *x*_*i*:*R*_*l*__ are shown in Table 2. Also, the 95% MLPI and BPI of *x*_*i*:*R*_*l*__ are summarized in Table 3.  Table 4 shows the 90% MLPI and BPI of FOS *y*_*s*_. The 95% MLPI and BPI of FOS *y*_*s*_ are listed in Table 5. The results of 90% MLPI and BPI of FURS *z*_*s*_ are given in Table 6. Also, the 95% MLPI and BPI of FURS *z*_*s*_ are obtained in Table 7.

**Figure 3:**
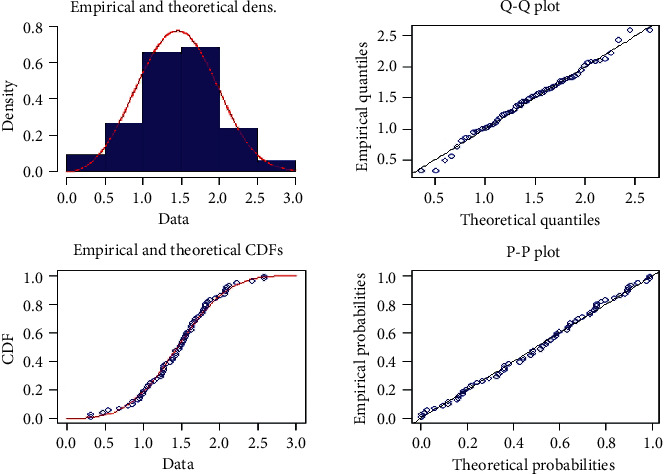
Empirical, Q-Q, and P-P plots of NWPD for real-life data.

**Example 2 .**

(Real-life data): The data are represented by the strength data measured in GPA, for single carbon fibres, and impregnated 1000 carbon fibre tows. For analyzed purposes, we consider single fibres of 20 mm with sample sizes *n*=67. These data are reported by Badar and Priest [25] and used by Kundu and Raqab [26]. The distance between the empirical and the fitted distribution functions as computed by using Kolmogorov-Smirnov (K-S) is 0.046121, and the corresponding *p* value is 0.9988 Since the *p* value is quite high, we cannot reject the null hypothesis that the data are coming from the NWPD. Empirical, *Q* − *Q*, and *P* − *P* plots are shown in Figure 3, which clear that the NWPD fits the data very well. The data are as follows:

(45) $$\begin{array}{c} \mathrm{6416806.fig-inline.001} \end{array}$$

The generated ProgT-II C sample from data set 1 with effective sample size *m*=30 and censoring scheme *R* = (5, 0, 0, 4, 0, 0, 3, 0, 0, 4, 0, 3, 0, 0, 3, 0, 0, 3, 0, 2, 0, 0, 2, 0, 0, 2, 0, 2, 0, 4) is given as follows: *x* = (0.312, 0.314, 0.479, 0.552, 0.70, 0.803, 0.861, 0.865, 0.944, 0.958, 0.966, 0.997, 1.006, 1.021, 1.055, 1.063, 1.098, 1.14, 1.179, 1.224, 1.240, 1.253, 1.270, 1.272, 1.274, 2.128, 2.233, 2.433, 2.585, 2.585). Based on the M-H technique within Gibbs sampling, we generate 32000 MCMC samples {(*δ*_*j*_, *β*_*j*_, *θ*_*j*_), *j*=1,2,…, 32000} and discard the first 2000 values as “burn-in” periods under the consideration of the noninformative prior gamma functions of *δ*, *β*, and *θ* with hyperparameters *γ*_*i*_ and *η*_*i*_=0, where *i*=1,2,3. The mean values of *δ*, *β*, and *θ* are given in Table 8. The results of 90% MLPI and BPI of *x*_*i*:*R*_*l*__ are shown in Table 9. Also, the 95% MLPI and BPI of *x*_*i*:*R*_*l*__ are summarized in Table 10.  Table 11 shows the 90% MLPI and BPI of FOS *y*_*s*_. The 95% MLPI and BPI of FOS *y*_*s*_ are listed in Table 12. The results of 90% MLPI and BPI of FURS *z*_*s*_ are shown in Table 13. Also, the 95% MLPI and BPI of FURS *z*_*s*_ are obtained in Table 14.

## 6. Conclusion

In this paper, we have dealt with OSP and TSP problems for future observations having an NWPD under the ProgT-II C sample. The predictions of FOS and FURS samples are also studied. The construction of PI for future unobserved failures in all cases is obtained based on the invariant property of MLEs and the generated MCMC samples. N-RI is considered a suitable numerical method used in our paper to get the bounds of PI. A simulated data set and a real-life data set are performed to demonstrate the discussed methods. Summing up the results, it can be concluded that

- It is clear from all tables that the length of the MLPI is smaller than the length of the BPI.
- For increasing the value of the survivor units in the same position of *x*_*i*,*R*_*l*__ in the case of OSP and *s* in the case of FOS or FURS, the length of the PI increases.
- It can be seen that the length of 90% PI is smaller than the length of 95% PI, which proved that when the significance level *γ* increases, then the interval length decreases.
- In the TSP problem, the lengths of FOS are smaller than ones of FURS.
- Regarding the discussed problem, we can predict the exceedances of the future flood peaks based on the currently available data. Also, we can predict the unobserved value of the exceedances due to the recorded ones.
- Finally, we can conclude that the proposed inference methods give consistent results.
- Sometimes, the available data could be affected by uncertainties and/or inaccuracies. Therefore, strictly speaking, a prediction system based on soft computing techniques and, in particular, on the latest generation fuzzy techniques would be needed, see Cacciola et al. [27] as future work.

## Figures and Tables

**Table 1:** Mean value of *δ*, *β*, and *θ*.

Parameter	ML	Bayes
*δ*	2.3588	2.3291
*β*	1.8157	1.8059
*θ*	2.8754	2.8586

**Table 2:** 90% MLPI and BPI of *x*_*i*,*R*_*l*__ for simulated data.

Point	MLPI		BPI	
	[LB_1_,UB_1_]	Length	[LB_1_,UB_1_]	Length
*x*_1,1_	[0.3258, 2.2552]	1.9294	[0.321, 2.3457]	2.0247
*x*_1,2_	[0.8763, 3.6822]	2.8059	[0.8678, 3.9746]	3.1068
*x*_4,1_	[0.5625, 3.3224]	2.7599	[0.5621, 3.5239]	2.9618
*x*_7,1_	[0.6729, 2.3546]	1.6818	[0.6733, 2.4587]	1.7854
*x*_7,2_	[1.0765, 3.7495]	2.673	[1.0779, 4.07]	2.9922
*x*_10,1_	[0.7472, 2.3824]	1.6353	[0.7478, 2.4891]	1.7413
*x*_10,2_	[1.1284, 3.7686]	2.6402	[1.1303, 4.0948]	2.9645
*x*_12,1_	[0.9798, 3.4471]	2.4672	[0.9813, 3.6738]	2.6925
*x*_15,1_	[0.9983, 2.4911]	1.4927	[0.9991, 2.6061]	1.607
*x*_15,2_	[0.5527, 3.8441]	3.2914	[0.5589, 4.189]	3.6301
*x*_20,1_	[1.3866, 2.6969]	1.3103	[1.3871, 2.8236]	1.4365
*x*_20,2_	[1.0821, 3.9911]	2.9091	[1.0841, 4.3632]	3.2791
*x*_23,1_	[1.666, 2.8681]	1.2021	[1.6664, 3.0023]	1.336
*x*_23,2_	[1.4116, 4.1171]	2.7054	[1.4156, 4.5071]	3.0915
*x*_25,1_	[1.9304, 3.0443]	1.1139	[1.9307, 3.1851]	1.2544
*x*_25,2_	[1.7086, 4.2499]	2.5413	[1.7146, 4.6557]	2.9412
*x*_27,1_	[2.0513, 3.1288]	1.0776	[2.0514, 3.2723]	1.2208
*x*_27,2_	[1.8413, 4.3145]	2.4733	[1.8481, 4.7272]	2.8791
*x*_30,1_	[2.5348, 3.4873]	0.9526	[2.5347, 3.6403]	1.1055
*x*_30,2_	[2.3606, 4.5957]	2.2351	[2.3704, 5.0329]	2.6626

**Table 3:** 95% MLPI and BPI of *x*_*i*,*R*_*l*__ for simulated data.

Point	MLPI		BPI	
	[LB_1_,UB_1_]	Length	[LB_1_,UB_1_]	Length
*x*_1,1_	[0.2704, 2.5258]	2.2554	[0.267, 2.6629]	2.3959
*x*_1,2_	[0.7217, 4.0511]	3.3294	[0.7095, 4.4978]	3.7883
*x*_4,1_	[0.493, 3.7171]	3.2241	[0.4924, 4.0377]	3.5453
*x*_7,1_	[0.6438, 2.6167]	1.9728	[0.644, 2.774]	2.1299
*x*_7,2_	[0.9492, 4.1134]	3.1643	[0.949, 4.5974]	3.6484
*x*_10,1_	[0.7206, 2.6422]	1.9216	[0.7209, 2.8035]	2.0826
*x*_10,2_	[1.0064, 4.1311]	3.1247	[1.0069, 4.6228]	3.6159
*x*_12,1_	[0.9371, 3.8311]	2.894	[0.9378, 4.1877]	3.2498
*x*_15,1_	[0.9775, 2.7424]	1.7649	[0.9778, 2.9167]	1.9388
*x*_15,2_	[0.6822, 4.2013]	3.519	[0.6846, 4.7183]	4.0337
*x*_20,1_	[1.3707, 2.9337]	1.563	[1.371, 3.1267]	1.7557
*x*_20,2_	[1.1617, 4.3384]	3.1767	[1.1629, 4.8926]	3.7297
*x*_23,1_	[1.6523, 3.094]	1.4417	[1.6525, 3.2993]	1.6468
*x*_23,2_	[1.4764, 4.4562]	2.9798	[1.4791, 5.0355]	3.5565
*x*_25,1_	[1.9183, 3.2602]	1.3419	[1.9184, 3.4761]	1.5577
*x*_25,2_	[1.7643, 4.5809]	2.8166	[1.7684, 5.1824]	3.414
*x*_27,1_	[2.0397, 3.3402]	1.3005	[2.0398, 3.5606]	1.5208
*x*_27,2_	[1.8938, 4.6418]	2.748	[1.8984, 5.2529]	3.3544
*x*_30,1_	[2.5251, 3.6817]	1.1566	[2.525, 3.9179]	1.3928
*x*_30,2_	[2.4037, 4.9075]	2.5039	[2.4104, 5.5536]	3.1433

**Table 4:** 90% MLPI and BPI of FOS *y*_*s* _ for simulated data.

Point	MLPI		BPI	
	[LB_2_,UB_2_]	Length	[LB_2_,UB_2_]	Length
*y*_1_	[0.0982, 0.9228]	0.8246	[0.0835, 0.9609]	0.8774
*y*_2_	[0.2937, 1.2252]	0.9315	[0.2632, 1.2762]	1.013
*y*_3_	[0.4797, 1.4809]	1.0012	[0.4426, 1.5462]	1.1036
*y*_4_	[0.659, 1.7253]	1.0662	[0.6202, 1.8094]	1.1892
*y*_5_	[0.8383, 1.9747]	1.1365	[0.8002, 2.0847]	1.2845
*y*_6_	[1.0238, 2.2435]	1.2196	[0.9876, 2.3894]	1.4018
*y*_7_	[1.2234, 2.5502]	1.3268	[1.1889, 2.747]	1.5581
*y*_8_	[1.4488, 2.9288]	1.48	[1.4144, 3.2]	1.7855
*y*_9_	[1.7232, 3.4613]	1.7381	[1.6857, 3.8514]	2.1657
*y*_10_	[2.1158, 4.4795]	2.3636	[2.0678, 5.1097]	3.0419

**Table 5:** 95% MLPI and BPI of FOS *y*_*s* _ for simulated data.

Point	MLPI		BPI	
	[LB_2_,UB_2_]	Length	[LB_2_,UB_2_]	Length
*y*_1_	[0.0666, 1.0349]	0.9683	[0.0536, 1.0833]	1.0297
*y*_2_	[0.2378, 1.3389]	1.1012	[0.2054, 1.4039]	1.1986
*y*_3_	[0.4113, 1.5984]	1.187	[0.3688, 1.6833]	1.3145
*y*_4_	[0.5817, 1.8479]	1.2662	[0.5352, 1.9597]	1.4245
*y*_5_	[0.7531, 2.1042]	1.3511	[0.7062, 2.253]	1.5468
*y*_6_	[0.9309, 2.3823]	1.4514	[0.8854, 2.5821]	1.6967
*y*_7_	[1.1218, 2.7024]	1.5806	[1.0783, 2.9738]	1.8955
*y*_8_	[1.3364, 3.102]	1.7655	[1.2941, 3.4778]	2.1837
*y*_9_	[1.5956, 3.6738]	2.0782	[1.5516, 4.2163]	2.6647
*y*_10_	[1.9599, 4.7998]	2.8399	[1.9072, 5.6859]	3.7788

**Table 6:** 90% MLPI and BPI of FURS *z*_*s* _ for simulated data.

Point	MLPI		BPI	
	[LB_3_,UB_3_]	Length	[LB_3_,UB_3_]	Length
*z*_1_	[0.3491, 3.28]	2.9309	[0.332, 3.4623]	3.1303
*z*_2_	[1.0138, 4.225]	3.2112	[1.0004, 4.6282]	3.6278
*z*_3_	[1.6044, 4.9377]	3.3333	[1.5923, 5.5943]	4.0019
*z*_4_	[2.1286, 5.5379]	3.4093	[2.103, 6.4689]	4.3659
*z*_5_	[2.604, 6.068]	3.464	[2.5488, 7.2876]	4.7388
*z*_6_	[3.0422, 6.5489]	3.5067	[2.9434, 8.0667]	5.1233
*z*_7_	[3.4511, 6.9927]	3.5417	[3.2973, 8.8153]	5.518
*z*_8_	[3.836, 7.4074]	3.5713	[3.6185, 9.5392]	5.9207
*z*_9_	[4.2011, 7.7982]	3.5971	[3.9128, 10.2422]	6.3293
*z*_10_	[4.5492, 8.1691]	3.6198	[4.185, 10.927]	6.742

**Table 7:** 95% MLPI and BPI of FURS *z*_*s* _ for simulated data.

Point	MLPI		BPI	
	[LB_3_,UB_3_]	Length	[LB_3_,UB_3_]	Length
*z*_1_	[0.2367, 3.6784]	3.4418	[0.2183, 3.9734]	3.755
*z*_2_	[0.8209, 4.6163]	3.7955	[0.8006, 5.2348]	4.4342
*z*_3_	[1.3759, 5.3265]	3.9506	[1.3594, 6.3007]	4.9413
*z*_4_	[1.8794, 5.9256]	4.0462	[1.8544, 7.277]	5.4226
*z*_5_	[2.3407, 6.4552]	4.1145	[2.2909, 8.1982]	5.9073
*z*_6_	[2.7685, 6.9359]	4.1675	[2.6784, 9.0806]	6.4023
*z*_7_	[3.1691, 7.3798]	4.2107	[3.0254, 9.9332]	6.9078
*z*_8_	[3.5474, 7.7946]	4.2472	[3.3392, 10.7615]	7.4224
*z*_9_	[3.9069, 8.1857]	4.2787	[3.6254, 11.5695]	7.9441
*z*_10_	[4.2502, 8.5568]	4.3066	[3.8888, 12.3599]	8.4711

**Table 8:** Mean value of *δ*, *β*, and *θ* for real-life data.

Parameter	ML	Bayes
*δ*	2.3535	2.4621
*β*	1.9859	1.9742
*θ*	3.1472	3.2208

**Table 9:** 90% MLPI and BPI of *x*_*i*,*R*_*l*__ for real-life data.

Point	MLPI		BPI	
	[LB_1_,UB_1_]	Length	[LB_1_,UB_1_]	Length
*x*_1,1_	[0.3735, 1.6116]	1.2382	[0.3715, 1.6611]	1.2897
*x*_1,2_	[0.6524, 2.141]	1.4885	[0.6433, 2.2324]	1.5891
*x*_1,3_	[0.9836, 2.6626]	1.679	[0.9727, 2.8196]	1.8469
*x*_1,4_	[1.3579, 3.3059]	1.948	[1.3468, 3.5738]	2.227
*x*_1,5_	[1.8516, 4.4145]	2.5628	[1.8357, 4.9122]	3.0765
*x*_4,1_	[0.5981, 1.8543]	1.2561	[0.5981, 1.9147]	1.3167
*x*_4,2_	[0.855, 2.4798]	1.6248	[0.8531, 2.6043]	1.7512
*x*_4,3_	[1.2227, 3.1789]	1.9562	[1.2194, 3.4125]	2.1931
*x*_4,4_	[1.7267, 4.331]	2.6042	[1.7187, 4.7944]	3.0757
*x*_7,1_	[0.9014, 2.2211]	1.3197	[0.902, 2.3033]	1.4014
*x*_7,2_	[0.5061, 3.0276]	2.5215	[0.5167, 3.2189]	2.7022
*x*_7,3_	[1.6322, 4.2414]	2.6092	[1.6304, 4.6643]	3.0339
*x*_10,1_	[0.9855, 2.0148]	1.0293	[0.986, 2.0805]	1.0945
*x*_10,2_	[1.1604, 2.6024]	1.4421	[1.1615, 2.7413]	1.5798
*x*_10,3_	[1.4536, 3.2759]	1.8222	[1.4526, 3.5317]	2.0792
*x*_10,4_	[1.8979, 4.4029]	2.505	[1.8881, 4.8949]	3.0068
*x*_12,1_	[1.0321, 2.2779]	1.2458	[1.0327, 2.3628]	1.3301
*x*_12,2_	[0.7123, 3.0697]	2.3575	[0.7157, 3.2671]	2.5514
*x*_12,3_	[1.7084, 4.2717]	2.5633	[1.706, 4.7036]	2.9976
*x*_15,1_	[1.0883, 2.3042]	1.2159	[1.0889, 2.3902]	1.3013
*x*_15,2_	[0.7911, 3.0894]	2.2983	[0.7934, 3.2893]	2.496
*x*_15,3_	[1.7432, 4.2859]	2.5427	[1.7403, 4.7216]	2.9813
*x*_18,1_	[1.1709, 2.3448]	1.1738	[1.1715, 2.4323]	1.2608
*x*_18,2_	[0.9009, 3.1198]	2.2189	[0.9023, 3.3236]	2.4212
*x*_18,3_	[1.7962, 4.308]	2.5118	[1.7926, 4.7493]	2.9567
*x*_20,1_	[1.2671, 2.7944]	1.5273	[1.2679, 2.923]	1.6551
*x*_20,2_	[0.8101, 4.1296]	3.3195	[0.813, 4.4941]	3.681
*x*_23,1_	[1.3116, 2.8151]	1.5035	[1.3123, 2.9449]	1.6325
*x*_23,2_	[0.8777, 4.1438]	3.266	[0.8801, 4.5108]	3.6307
*x*_26,1_	[2.1533, 3.2961]	1.1428	[2.1535, 3.4449]	1.2914
*x*_26,2_	[1.9179, 4.486]	2.5681	[1.925, 4.8971]	2.9721
*x*_28,1_	[2.4552, 3.5019]	1.0467	[2.4552, 3.6557]	1.2005
*x*_28,2_	[2.2512, 4.6399]	2.3888	[2.2601, 5.0641]	2.804
*x*_30,1_	[2.5955, 3.1388]	0.5434	[2.5955, 3.2208]	0.6254
*x*_30,2_	[2.5164, 3.5468]	1.0303	[2.5204, 3.7297]	1.2094
*x*_30,3_	[2.8091, 4.0685]	1.2594	[2.7972, 4.3976]	1.6004
*x*_30,4_	[2.2466, 5.0227]	2.7761	[2.2995, 5.6178]	3.3183

**Table 10:** 95% MLPI and BPI of *x*_*i*,*R*_*l*__ for real-life data.

Point	MLPI		BPI	
	[LB_1_,UB_1_]	Length	[LB_1_,UB_1_]	Length
*x*_1,1_	[0.3437, 1.7832]	1.4395	[0.3425, 1.851]	1.5085
*x*_1,2_	[0.5664, 2.3197]	1.7533	[0.5565, 2.4483]	1.8918
*x*_1,3_	[0.8684, 2.8581]	1.9897	[0.8541, 3.0816]	2.2275
*x*_1,4_	[1.2182, 3.536]	2.3178	[1.203, 3.918]	2.715
*x*_1,5_	[1.6771, 4.7418]	3.0647	[1.6584, 5.4544]	3.796
*x*_4,1_	[0.5752, 2.0415]	1.4663	[0.5752, 2.1272]	1.5521
*x*_4,2_	[0.7713, 2.6823]	1.911	[0.7687, 2.8611]	2.0924
*x*_4,3_	[1.0966, 3.4153]	2.3187	[1.0912, 3.7513]	2.6601
*x*_4,4_	[1.5561, 4.6638]	3.1077	[1.5462, 5.3321]	3.7859
*x*_7,1_	[0.8812, 2.4307]	1.5495	[0.8814, 2.5514]	1.67
*x*_7,2_	[0.6232, 3.2708]	2.6476	[0.6276, 3.5499]	2.9223
*x*_7,3_	[1.4789, 4.5802]	3.1012	[1.4762, 5.1958]	3.7196
*x*_10,1_	[0.9717, 2.1886]	1.2169	[0.9719, 2.2837]	1.3118
*x*_10,2_	[1.0999, 2.7962]	1.6964	[1.1004, 2.9956]	1.8952
*x*_10,3_	[1.349, 3.5058]	2.1569	[1.3471, 3.8717]	2.5246
*x*_10,4_	[1.7439, 4.7307]	2.9869	[1.7336, 5.4356]	3.702
*x*_12,1_	[1.0145, 2.4828]	1.4683	[1.0148, 2.6079]	1.5931
*x*_12,2_	[0.8001, 3.3099]	2.5098	[0.8016, 3.5975]	2.7959
*x*_12,3_	[1.5626, 4.6083]	3.0457	[1.5595, 5.2357]	3.6762
*x*_15,1_	[1.0716, 2.507]	1.4354	[1.0719, 2.6339]	1.562
*x*_15,2_	[0.8711, 3.3281]	2.457	[0.8721, 3.6194]	2.7473
*x*_15,3_	[1.6004, 4.6214]	3.021	[1.5971, 5.2539]	3.6568
*x*_18,1_	[1.1554, 2.5443]	1.389	[1.1556, 2.6739]	1.5183
*x*_18,2_	[0.9721, 3.3564]	2.3844	[0.9727, 3.653]	2.6803
*x*_18,3_	[1.658, 4.642]	2.984	[1.6541, 5.2818]	3.6277
*x*_20,1_	[1.2454, 3.0457]	1.8002	[1.2458, 3.2397]	1.9939
*x*_20,2_	[0.9409, 4.4757]	3.5348	[0.942, 5.0156]	4.0736
*x*_23,1_	[1.2907, 3.0647]	1.774	[1.2911, 3.2607]	1.9696
*x*_23,2_	[0.9998, 4.4888]	3.4889	[1.0008, 5.0322]	4.0314
*x*_26,1_	[2.1405, 3.5121]	1.3716	[2.1406, 3.7391]	1.5985
*x*_26,2_	[1.9774, 4.8068]	2.8295	[1.9821, 5.4112]	3.4291
*x*_28,1_	[2.444, 3.706]	1.2621	[2.444, 3.9417]	1.4977
*x*_28,2_	[2.3021, 4.9509]	2.6488	[2.3082, 5.5739]	3.2657
*x*_30,1_	[2.5902, 3.2537]	0.6636	[2.5902, 3.3793]	0.7891
*x*_30,2_	[2.5354, 3.6919]	1.1565	[2.5381, 3.9578]	1.4197
*x*_30,3_	[2.7559, 4.2564]	1.5005	[2.7454, 4.7241]	1.9787
*x*_30,4_	[2.2953, 5.3129]	3.0176	[2.3393, 6.1543]	3.815

**Table 11:** 90% MLPI and BPI of FOS *y*_*s* _ for real-life data.

Point	MLPI		BPI	
	[LB_2_,UB_2_]	Length	[LB_2_,UB_2_]	Length
*y*_1_	[0.1438, 1.1146]	0.9709	[0.1273, 1.1518]	1.0245
*y*_2_	[0.3913, 1.4443]	1.053	[0.3618, 1.4965]	1.1347
*y*_3_	[0.6128, 1.7176]	1.1048	[0.5796, 1.7875]	1.2079
*y*_4_	[0.8193, 1.975]	1.1557	[0.7862, 2.0681]	1.2818
*y*_5_	[1.0209, 2.2346]	1.2137	[0.9895, 2.3586]	1.3691
*y*_6_	[1.2257, 2.5111]	1.2854	[1.1959, 2.6765]	1.4807
*y*_7_	[1.4424, 2.8233]	1.3809	[1.413, 3.0451]	1.6321
*y*_8_	[1.6835, 3.2041]	1.5205	[1.652, 3.5052]	1.8532
*y*_9_	[1.9729, 3.7328]	1.7599	[1.9349, 4.1549]	2.22
*y*_10_	[2.3801, 4.7253]	2.3451	[2.3275, 5.3778]	3.0503

**Table 12:** 95% MLPI and BPI of FOS *y*_*s* _ for real-life data.

Point	MLPI		BPI	
	[LB_2_,UB_2_]	Length	[LB_2_,UB_2_]	Length
*y*_1_	[0.1007, 1.2378]	1.1371	[0.0855, 1.2853]	1.1998
*y*_2_	[0.3226, 1.5664]	1.2439	[0.2901, 1.634]	1.3439
*y*_3_	[0.5324, 1.8418]	1.3093	[0.4931, 1.9342]	1.441
*y*_4_	[0.731, 2.103]	1.372	[0.6902, 2.2283]	1.5381
*y*_5_	[0.9256, 2.3682]	1.4425	[0.886, 2.5373]	1.6513
*y*_6_	[1.1235, 2.6528]	1.5292	[1.0856, 2.8801]	1.7944
*y*_7_	[1.3325, 2.9769]	1.6444	[1.2956, 3.2827]	1.9871
*y*_8_	[1.5638, 3.3769]	1.8131	[1.5258, 3.7926]	2.2668
*y*_9_	[1.8389, 3.9418]	2.1029	[1.7957, 4.5254]	2.7297
*y*_10_	[2.2192, 5.0332]	2.814	[2.1626, 5.9429]	3.7802

**Table 13:** 90% MLPI and BPI of FURS *z*_*s* _ for real-life data.

Point	MLPI		BPI	
	[LB_3_,UB_3_]	Length	[LB_3_,UB_3_]	Length
*z*_1_	[0.4583, 3.5537]	3.0954	[0.4426, 3.7525]	3.3099
*z*_2_	[1.2147, 4.4792]	3.2645	[1.2056, 4.9012]	3.6956
*z*_3_	[1.8481, 5.1653]	3.3172	[1.8378, 5.833]	3.9952
*z*_4_	[2.3933, 5.7365]	3.3432	[2.3651, 6.6633]	4.2982
*z*_5_	[2.8776, 6.2365]	3.3589	[2.8162, 7.4304]	4.6143
*z*_6_	[3.3173, 6.6869]	3.3696	[3.2101, 8.1524]	4.9423
*z*_7_	[3.7227, 7.1001]	3.3774	[3.5601, 8.8395]	5.2795
*z*_8_	[4.1007, 7.4841]	3.3834	[3.8753, 9.4982]	5.6229
*z*_9_	[4.4561, 7.8443]	3.3883	[4.1625, 10.1329]	5.9704
*z*_10_	[4.7925, 8.1847]	3.3922	[4.4269, 10.7469]	6.32

**Table 14:** 95% MLPI and BPI of FURS *z*_*s* _ for real-life data.

Point	MLPI		BPI	
	[LB_3_,UB_3_]	Length	[LB_3_,UB_3_]	Length
*z*_1_	[0.3212, 3.9464]	3.6252	[0.3031, 4.2632]	3.9601
*z*_2_	[1.0015, 4.8571]	3.8556	[0.986, 5.4956]	4.5096
*z*_3_	[1.6059, 5.536]	3.93	[1.5927, 6.5158]	4.923
*z*_4_	[2.1358, 6.1026]	3.9668	[2.1097, 7.4355]	5.3258
*z*_5_	[2.6104, 6.5994]	3.989	[2.555, 8.2922]	5.7372
*z*_6_	[3.0434, 7.0474]	4.004	[2.9444, 9.1036]	6.1592
*z*_7_	[3.4437, 7.4586]	4.015	[3.2896, 9.8799]	6.5903
*z*_8_	[3.8177, 7.841]	4.0234	[3.5995, 10.6276]	7.0281
*z*_9_	[4.1699, 8.1999]	4.03	[3.8806, 11.3512]	7.4706
*z*_10_	[4.5037, 8.5392]	4.0355	[4.1381, 12.0541]	7.916

## Data Availability

All the relevant data are within the paper.

## Acronyms

BPI: Bayesian predictive interval
NWPD: New Weibull-Pareto distribution
FOS: Future-order statistic
PI: Predictive interval
FURS: Future upper record statistic
PPE: Predictive posterior expectation
MCMC: Markov chain Monte Carlo
ProgT-II C: Progressive type-II censoring
M-H: Metropolis-Hastings
OSP: One-sample prediction
ML: Maximum likelihood
TSP: Two-sample prediction
MLPI: ML predictive interval
[LB,UB]: [Lower bound, Upper bound].

## References

[1] Ghafouri S., Habibi Rad A., Yousefzadeh F. (2015). Two-sample prediction for progressively Type-II censored Weibull lifetimes. *Communications in Statistics-Simulation and Computation*.

[2] Pushpalatha M. N., Parkavi A., Alex S. A. (2022). Predictive analytics for healthcare. *Deep Learning Applications for Cyber-Physical*.

[3] Lee S. C., Cheang S. Y. P., Moslehpour M. (2022). Predictive analytics in business analytics: decision tree. *Advances in Decision Sciences*.

[4] Burnaev E. (2019). Algorithmic foundations of predictive analytics in industrial engineering design. *Journal of Communications Technology and Electronics*.

[5] Sharma A., Vijayakumar V. (2018). Predictive analytics in weather forecasting using machine learning algorithms. *EAI Endorsed Transactions on Cloud Systems*.

[6] Asher L. A., Clinton J., Devin J. C., Bydon M. (2018). Introduction. Predictive analytics in medicine. *Neurosurgical Focus*.

[7] Suleman N., Albert L. (2015). The new Weibull-pareto distribution. *Pakistan Journal of Statistics and Operation Research*.

[8] Ghafouri S., Habibi Rad A., Doostparast M. (2011). Bayesian two-sample prediction with progressively Type-II censored data for some lifetime models. *Journal of the Iranian Statistical Society*.

[9] Abdel-Hamid A. H. (2016). Properties, estimations and predictions for a Poisson-Half-Logistic distribution based on progressively Type-II censored samples. *Applied Mathematical Modelling*.

[10] Al-Hussaini E. K., Abdel-Hamid A. H., Hashem A. F. (2014). Bayesian prediction intervals of order statistics based on progressively type-II censored competing risks data from the half-logistic distribution. *Journal of the Egyptian Mathematical Society*.

[11] Raqab M. Z., Asgharzadeh A., Valiollahi R. (2010). Prediction for Pareto distribution based on progressively Type-II censored samples. *Computational Statistics & Data Analysis*.

[12] Golparvar L., Parsian A. (2014). On Bayes predictor of times to failure of Type-II progressively censored sample. *Journal of Statistical Computation and Simulation*.

[13] Soliman A. A., Abd Ellah A. H., Abou-Elheggag N. A., El-Sagheer R. M. (2013). Bayesian and frequentist prediction using progressive type-II censored with binomial removals. *Intelligent Information Management*.

[14] Mahmoud M. A. W., Soliman A. A., Abd Ellah A. H., El-Sagheer R. M. (2013). Bayesian inference and prediction using progressive first-failure censored from generalized Pareto distribution. *Journal of Statistics Applications & Probability*.

[15] El-Sagheer R. M. (2016). Bayesian prediction based on general progressive censored data from generalized Pareto distribution. *Journal of Statistics Applications & Probability*.

[16] Ahmed E. A. (2016). Estimation and prediction for the generalized inverted exponential distribution based on progressively first-failure-censored data with application. *Journal of Applied Statistics*.

[17] Abushal T. A., Al-Zaydi A. M. (2012). Prediction based on generalized order statistics from a mixture of Rayleigh distributions using MCMC algorithm. *Open Journal of Statistics*.

[18] Abushal T. A., Al-Zaydi A. M. (2012). Bayesian prediction based on generalized order statistics from a mixture of two exponentiated Weibull distribution via MCMC simulation. *International Journal of Statistics and Probability*.

[19] Balakrishnan N., Aggarwala R. (2000). *Progressive Censoring: Theory, Methods and Applications*.

[20] El-Sagheer R. M. (2018). Estimation of parameters of Weibull–Gamma distribution based on progressively censored data. *Statistical Papers*.

[21] Mahmoud M. A. W., El-Sagheer R. M., Abdallah S. H. M. (2016). Inferences for new Weibull-Pareto distribution based on progressively Type-II censored data. *Journal of Statistics Applications & Probability*.

[22] David H. A., Nagaraja H. N. (2003). *Order Statistics*.

[23] Chandler K. N. (1952). The distribution and frequency of record values. *Journal of the Royal Statistical Society: Series B*.

[24] Balakrishnan N., Sandhu R. A. (1995). A simple simulation algorithm for generating progressively type-II censored samples. *The American Statistician*.

[25] Bader M. G., Priest A. M., Hayashi T., Kawata K., Umekawa S. (1982). *Statistical Aspects of Fiber and Bundle Strength in Hybrid Composites. Progress in Science and Engineering Composites*.

[26] Kundu D., Raqab M. (2009). Estimation of *R*=*P*(*Y* > *X*)for three-parameter Weibull distribution. *Statistics & Probability Letters*.

[27] Cacciola M., Pellican D., Megali G., Lay-Ekuakille A., Versaci M., Morabito F. C. Aspects about air pollution prediction on urban environment.

